# Health Tract, No. 35

**Published:** 1861-01

**Authors:** 


### HEALTH TRACT, No. 35.

---

### DRINKING.

Man is the only animal that drinks without being thirsty, swallowing whole quarts of water when Nature does not call for it, with the alleged view of "washing out" the system. When persons are thirsty, that thirst should be fully assuaged with moderately cool water, drank (in summer time or under great bodily heat or fatigue) very leisurely, but not within half an hour of eating a regular meal. Eminent physioiogists agree that drinking at meals dilutes the gastric juice, diminishes its solvent power, and retards digestion, especially if what is drank is cold. Persons in vigorous health, and who work or exercise a great part of every day in the open air, may drink a glass of water, or a single cup of weak coffee or tea, at each meal, and live to a good old age. But it is very certain that sedentary persons and invalids can not go beyond that habitually, with impunity. The wisdom of such consists in drinking nothing at all at the regular meals beyond a swallow or two at a time of some hot drink of a mild and nutritious character. Feeble persons will be benefited by hot drinks, because they warm up the body, excite the circulation, and thus promote digestion, if taken while eating, and not exceeding a cupful.

Cold water ought never to be drank within half an hour of eating; for the colder it is, the more instantly does it arrest digestion, not only by diluting the gastric juice, but by reducing its temperature, which is near one hundred degrees. Ice-water is something over thirty-two degrees, and, when swallowed, mixes with the gastric juice, and lowers its temperature, not to be elevated until heat enough has been withdrawn from the general system; and that draft must be made until the hundred degrees of warmth are attained: but some persons have so little vitality, that the body exhausts itself in its instinctive efforts to help the stomach, from which its life and strength come; and the person rises from the table with a cold chill running down the back or over the whole body. Sometimes these drafts upon the body for warmth to the stomach are so sudden and great, that they can not be met, and instantaneous death is the result. Many a person has dropped dead at the pump or at the spring; such a result is more certain if, in addition to the person being very warm at the time of drinking, there is also great bodily fatigue. A French general recently fell dead from drinking cold water on reaching the top of a mountain over-heated and exhausted in the effort of bringing up his battalions with promptitude. Under all circumstances of heat or fatigue, the glass of water should be grasped in the hand, held half a minute, then, taking not over two swallows, rest a quarter of a minute; then two swallows more, and so on, until the thirst is *nearly* assuaged. It will seldom happen that a person is inclined to take over half a dozen swallows thus.

No case is remembered in the practice of a quarter of a century, where malt liquors, wines, brandies, or any alcoholic drinks whatever, have ever had a permanent good effect in improving the digestion. Apparent advantages sometimes result, but they are transient or deceptive. If there is no appetite, it is because Nature has provided no gastric juice; and that is the product of Nature, not of alcohol. If there is appetite but no digestive power, liquor no more supplies that power than would the lash give strength to an exhausted donkey. If torture does arouse the sinking beast, it is only that it shall fall a little later into a still greater exhaustion from which there is no recovery; so with the use of liquor and tobacco as whetters of the appetite, when at length the desire for the accustomed stimulus ceases, and the man "sickens;" there is no longer a relish for the dram and the chew, and life fades apace, either in a stupor from which there is no awaking, or by wasting and uncontrollable diarrhea.

